# Phenanthrene-Degrading and Nickel-Resistant *Neorhizobium* Strain Isolated from Hydrocarbon-Contaminated Rhizosphere of *Medicago sativa* L.

**DOI:** 10.3390/microorganisms12081586

**Published:** 2024-08-04

**Authors:** Sergey Golubev, Margarita Rasterkovskaya, Irina Sungurtseva, Andrey Burov, Anna Muratova

**Affiliations:** Institute of Biochemistry and Physiology of Plants and Microorganisms, Saratov Scientific Centre of the Russian Academy of Sciences (IBPPM RAS), Saratov 410049, Russia; sngolubev@rambler.ru (S.G.); margueriterasterkowski@gmail.com (M.R.); airinmind@yandex.ru (I.S.); burov.anmi@gmail.com (A.B.)

**Keywords:** *Neorhizobium*, PAHs, phenanthrene, microbial degradation, nickel resistance

## Abstract

Pollutant degradation and heavy-metal resistance may be important features of the rhizobia, making them promising agents for environment cleanup biotechnology. The degradation of phenanthrene, a three-ring polycyclic aromatic hydrocarbon (PAH), by the rhizobial strain Rsf11 isolated from the oil-polluted rhizosphere of alfalfa and the influence of nickel ions on this process were studied. On the basis of whole-genome and polyphasic taxonomy, the bacterium Rsf11 represent a novel species of the genus *Neorhizobium*, so the name *Neorhizobium phenanthreniclasticum* sp. nov. was proposed. Analysis of phenanthrene degradation by the Rsf1 strain revealed 1-hydroxy-2-naphthoic acid as the key intermediate and the activity of two enzymes apparently involved in PAH degradation. It was also shown that the nickel resistance of Rsf11 was connected with the extracellular adsorption of metal by EPS. The joint presence of phenanthrene and nickel in the medium reduced the degradation of PAH by the microorganism, apparently due to the inhibition of microbial growth but not due to the inhibition of the activity of the PAH degradation enzymes. Genes potentially involved in PAH catabolism and nickel resistance were discovered in the microorganism studied. *N. phenanthreniclasticum* strain Rsf11 can be considered as a promising candidate for use in the bioremediation of mixed PAH–heavy-metal contamination.

## 1. Introduction

Polycyclic aromatic hydrocarbons (PAHs) and heavy metals (HMs) are priority environmental pollutants [1]. The content of these substances in all natural objects, including soil, is subject to mandatory systematic monitoring and regulation due to their high toxicity [2,3]. Soil is the main reservoir for the accumulation of PAHs and HMs, which enter and accumulate in it directly, via precipitation, or as a result of the movement of water masses [4,5]. These pollutants are often found simultaneously in contaminated sites that are the result of either natural processes (such as volcanic eruptions, forest fires, etc.) or anthropogenic activities (such as coal and oil mining and refining or electronic waste) [6,7]. Oil pollution is one example of a combination of PAHs and HMs that produces all the resulting environmental risks [8,9,10]. The PAHs in crude oil are predominantly represented by compounds of 2–3 aromatic rings, and their content can reach 4000 mg kg^−1^ [11]. Together with vanadium, nickel is the most common “petroleum metal”, the content of which in crude oil can reach 340 ppm [10,12]. Some studies have shown that joint PAH and HM pollution has greater harmful effects on the environment than the presence of the same pollutants separately [13,14,15], and therefore, ways to eliminate them are urgently needed.

Methods for the biological remediation of contaminated soils using specialized highly active microorganisms remain the technologies of choice for the restoration of contaminated areas, and their improvement toward searching for new, promising remediating strains is relevant. Many bacteria have great potential for bioremediation, but most of them are capable of either degrading PAHs or exhibiting resistance to HMs. Reports on bacteria that are able to simultaneously cope with complex PAH and HM pollution are few [16,17,18], which indicates insufficient knowledge of such microorganisms and the need for further identification and the exploration of the bacterial potential for the bioremediation of complex contaminants [19].

The physiological and biochemical characteristics of bacteria of the Rhizobiaceae family, as well as the recent intensification of research aimed at studying the bioremediation potential of these bacteria [20,21], give reason to believe that representatives of rhizobia can become successful candidates for the bioremediation/phytoremediation of soil co-contaminated with PAHs and HMs. There is evidence of the utilization of PAHs by rhizobia [22,23,24], as well as the effectiveness of rhizobia in the bioremediation of soils contaminated with HMs [21].

The objective of this study was to characterize the degradation of three-ringed PAH phenanthrene by the rhizobial strain Rsf11 and to assess the influence of nickel ions on this process.

## 2. Materials and Methods

### 2.1. Microorganism

*Neorhizobium* sp. Rsf11 was isolated as a phenanthrene-degrading bacterium from the rhizosphere of alfalfa (*Medicago sativa* L.) grown in oil-contaminated soil. This strain is registered and stored in the Collection of Rhizosphere Microorganisms of the Institute of Biochemistry and Physiology of Plants and Microorganisms (WFCC no. 975, WDCM no. 1021, http://collection.ibppm.ru, accessed on 20 June 2024) as IBPPM 350.

### 2.2. Species Identification

Identification of the strain studied at the species level was carried out using a taxonogenomics approach which integrates whole-genome-based taxonomy with standard polyphasic taxonomy [25].

The 16S rRNA gene sequence analysis was performed on the GGDC web server (http://ggdc.dsmz.de/, accessed on 20 June 2024; [26]) using the DSMZ phylogenomics pipeline [27] adapted to single genes. A multiple sequence alignment was created with MUSCLE [28]. Maximum likelihood (ML) and maximum parsimony (MP) trees were inferred from the alignment with RAxML [29] and TNT [30], respectively. For ML, rapid bootstrapping in conjunction with the autoMRE bootstopping criterion [31] and a subsequent search for the best tree was used; for MP, 1000 bootstrapping replicates were used in conjunction with tree bisection and reconnection branch swapping and ten random sequence addition replicates. The sequences were checked for a compositional bias using the Χ^2^ test as implemented in PAUP* [32]. Pairwise sequence similarities were calculated according to the recommendations of [33].

The whole-genome-based taxonomic analysis was carried out on the Type (Strain) Genome Server (TYGS), a free bioinformatics platform with the support of such authoritative resource for prokaryotic nomenclature and classification as the List of Prokaryotic names with Standing in Nomenclature (LPSN) (https://tygs.dsmz.de/, accessed on 9 June 2024; [26,34]. Determination of the closest type strain genomes was conducted in two complementary ways. Firstly, the query genome was compared against all type strain genomes available in the TYGS database via the MASH algorithm, a fast approximation of intergenomic relatedness [35], and the ten type strains with the smallest MASH distances were chosen. Secondly, an additional set of ten closely related type strains was determined via the 16S rRNA gene sequence. The latest was extracted from the target genome by using RNAmmer [36] and it was subsequently BLASTed [37] against the 16S rRNA gene sequences of the TYGS type strains. This was used as a proxy to find the best 50 matching type strains (according to the bitscore) and to subsequently calculate precise distances using the Genome BLAST Distance Phylogeny approach (GBDP) with the algorithm ‘coverage’ and distance formula *d*_5_ [38]. These distances were finally used to determine the ten closest type strain genomes. All pairwise comparisons among the set of genomes were conducted using GBDP and accurate intergenomic distances inferred with the algorithm ‘trimming’ and distance formula *d*_5_ [38]. Digital DNA–DNA hybridization (dDDH) values and confidence intervals were calculated using the recommended settings of the GGDC 4.0 [26,38]. In addition, online average nucleotide identity (ANI) calculation was performed at https://www.ezbiocloud.net/tools/ani (accessed on 26 June 2024) using the OrthoANIu algorithm [39]. The resulting intergenomic distances were used to infer a balanced minimum evolution tree with branch support via FASTME 2.1.6.1, including SPR postprocessing [40]. Branch support was inferred from 100 pseudo-bootstrap replicates each. The trees were rooted at the midpoint [41] and visualized with PhyD3 [42]. Species and subspecies clustering was conducted using 70% [34] and 79% [27] dDDH thresholds, respectively.

### 2.3. Cultural, Morphological, Physiological, and Biochemical Characteristics

Colony properties of the strain Rsf11 were observed on Yeast Mannitol Agar (YMA) medium. Cell morphology was examined by light and transmission electron microscopy. Microscopy of Gram-stained bacterial preparations was performed using a Leica DM2500 light microscope (Leica Microsystems GmbH, Wetzlar, Germany). Transmission electron microscopy (TEM) images were recorded on a Libra-120 transmission electron microscope (Carl Zeiss, Oberkochen, Germany) with an accelerating voltage of 120 kV at the Simbioz Center for the Collective Use of Research Equipment in the Field of Physico–Chemical Biology and Nanobiotechnology, IBPPM RAS, Saratov. Copper grids coated with a formvar film were used as substrates. Applied to the substrate was 5 µL of an aqueous suspension of bacteria, which was then kept for 30 min. Bacteria were characterized by size using the iTEM application.

The physiological characteristics were studied by standard methods using the Luria–Bertani (LB) and YMA media for bacterial cultivation [43]. Biochemical tests were conducted according to [44,45] and using the API^®^20NE test set (bioMerieux SA, Marcy l’Étoile, France).

### 2.4. DNA Extraction

The genomic DNA of a pure bacterial culture growing on the LB medium was extracted with a FastDNA SPIN kit (MP Biomedicals, Santa Ana, CA, USA). The quantity of extracted DNA was measured with a Qubit 2.0 fluorometer using a Qubit dsDNA HS assay kit.

### 2.5. DNA Library Preparation

Genomic DNA was sheared with a Covaris S220 ultrasonicator (Woburn, MA, USA). The DNA library was constructed with a NEBNext Ultra II DNA Library Prep Kit for Illumina according to the manufacturer’s instructions (San Diego, CA, USA). DNA quality was checked with a Bioanalyzer 2100 instrument (Agilent, Waldbronn, Germany) and an Agilent DNA high-sensitivity kit.

### 2.6. Whole-Genome Sequencing, Assembly and Annotation

The genome of the target strain was sequenced on an Illumina MiSeq platform in paired-end 300 bp mode. Sequence reads were preprocessed with the fastp v. 0.23.2 tool [46]. The genome was assembled de novo with SPAdes v. 3.15.5 [47]. This whole-genome shotgun project has been deposited at GenBank under the accession JBEAAL000000000 (BioProject ID, PRJNA1117657; BioSample ID, SAMN41578736; Assembly ID, GCA_040066075.1). The version described in this paper is JBEAAL010000000. The total features of the genome assembly are summarized in Table S1. Genome annotation was performed using the NCBI Prokaryotic Genome Annotation Pipeline (PGAP) (www.ncbi.nlm.nih.gov/genome/annotation_prok/, accessed on 30 May 2024), as well as the Prokka 1.14.6 tool [48]. Additionally, functional and pathway analysis with KEGG ORTHOLOGY (KO) assignment and KEGG mapping was carried out on the Blast KEGG Orthology and Links Annotation (BlastKOALA) web resource (https://www.kegg.jp/blastkoala/, accessed on 21 June 2024) [49]. For some individual genes/proteins, KO identifiers were assigned by direct search in the KO database located at https://www.genome.jp/kegg/ko.html (accessed on 23 June 2024) [50].

### 2.7. 16S rRNA Gene Amplification and Sequencing

PCR amplification of the16S rRNA gene and subsequent sequencing of the obtained PCR products using an ABI 3130xl genetic analyzer were conducted as described previously [51]. The 16S rRNA gene sequence has been deposited at GenBank under the accession OR826142.

### 2.8. Nickel Resistance

The maximum tolerant and minimum inhibitory concentrations (MTC and MIC) of nickel ions in the cultivation medium were assessed to define the resistance of strain Rsf11 to nickel. The MTC value was determined as the last concentration of nickel in the medium in which microorganism growth was observed, and the MIC value was determined as the minimum concentration of the metal in the medium in which there was no growth of the microorganism. Cultivation of strain Rsf11 was carried out in test tubes with 5 mL of LB medium supplemented with nickel (in the form of NiSO_4_ × 7H_2_O) at different concentrations (0, 0.2, 0.5, 1, 2, 3, 4, or 5 mM) at 29 °C for 5 days. Bacterial growth was determined turbidimetrically at 600 nm (Evolution 60, Thermo Scientific, Waltham, MA, USA) by determining the MIC and MTC of nickel.

The study of the extracellular adsorption and intracellular accumulation of nickel was carried out by determining the concentration of metal ions using the atomic adsorption spectroscopy method in the washout of the extracellular polymer substance, as well as in the biomass of washed cells according to [52]. Measurements were made on a Thermo Scientific iCE 3500 atomic absorption spectrometer (Thermo Scientific, USA).

### 2.9. Degradation of Phenanthrene in the Presence of Nickel

The ability of strain Rsf11 to degrade phenanthrene was assessed by cultivating the microorganism in MSM medium [24] containing phenanthrene as a sole carbon and energy source. Phenanthrene in an isopropanol solution (20 g L^−1^) was added to empty 0.2 L Erlenmeyer flasks to achieve a final concentration of 0.2 g L^−1^, and after the evaporation of the solvent, the MSM medium (50 mL) was added to the flasks. The degradation of phenanthrene in the presence of nickel was assessed by cultivating Rsf11 in MSM medium containing sodium glutamate (1 g L^−1^) and supplemented with phenanthrene (0.2 g L^−1^) and NiSO_4_ × 7H_2_O at different concentrations (0, 0.2, 0.5, 1 mM). Incubation was performed at 29 °C with rotary shaking (130 rpm at New Brunswick^TM^ Excella^®^ E24, Eppendorf, Hamburg, Germany) for 14 days. The control flasks contained the same medium and equal concentrations of phenanthrene and nickel without the inoculum. Phenanthrene degradation was determined by the elimination of PAH from the medium after incubation with the microorganism.

### 2.10. HPLC Analyses of Phenanthrene and Its Metabolites

The residual concentration of phenanthrene in the medium was analyzed by extraction with chloroform (5 mL per 50 mL of medium, three times for 15 min each) on a shaker. Extracts were combined, dried by solvent evaporation, and redissolved in acetonitrile for further analysis by high-performance liquid chromatography (HPLC). To analyze the phenanthrene degradation products, after the extraction of the culture liquid with chloroform, the metabolites were extracted with ethyl acetate (10 mL per 50 mL of medium, three times for 5 min each), and the extracts were combined and left until the solvent evaporated (neutral fraction). Then, the culture liquid was acidified with 1 M HCl to pH 2–3 and again extracted with ethyl acetate (10 mL per 50 mL of medium, three times for 5 min each), and the extracts were combined and left until the solvent evaporated (acidic fraction). A number of standard compounds were used to identify intermediate degradation metabolites: phenanthrene (≥99%, Aldrich, Saint Louis, MO, USA), 9-phenanthrol (tech., Aldrich), 9,10-phenanthrenequinone (≥99%, Aldrich), 1-hydroxy-2-naphthoic acid (>97%, Fluka, Basel, Switzerland), 2-carboxybenzaldehyde (97%, Aldrich), phthalic acid (pure grade, Reakhim, Moscow, Russia), salicylic acid (pharm., Reakhim), and 2,2-diphenic acid (>95%, Fluka). The metabolites were analyzed by HPLC.

HPLC analysis of phenanthrene and its microbial metabolites was carried out on an Agilent Technologies 1220 Infinity II LC chromatograph (Agilent Technology, Waldbronn, Germany) equipped with a 4.6 × 150 mm ZORBAX Eclipse PAH 5-Micron column (for phenanthrene determination) or a 4.6 × 150 mm ZORBAX Eclipse Plus C18 5-Micron column (for metabolite determination) and a 254 nm UV detector. For phenanthrene determination, the solvent system was H_2_O: acetonitrile, the linear gradient was 40–100% acetonitrile, and the time for the determination of residual phenanthrene was 17 min. For metabolite determination, the solvent system was H_2_O (acidified to pH 2.5): acetonitrile, the linear gradient was 40–100% acetonitrile, and the metabolite determination time was 17 min. PAHs and their metabolites were analyzed by comparing the retention times with those of standard compounds.

### 2.11. Enzyme Assays

To assess the activity of enzymes involved in the phenanthrene degradation, bacteria were grown on R2A medium containing 50 mg L^−1^ phenanthrene for 3 days. The microbial biomass was scraped from the surface of the medium and resuspended in 0.05 M Na-P buffer (pH 7.0). The cells were centrifuged (12,000× *g*, 10 min, Eppendorf Centrifuge 5810), then washed twice with 0.05 M Tris-HCl buffer (pH 7.5) with repeated centrifugation. The cell biomass was resuspended in the same buffer and disrupted by ultrasonic treatment (five times for 20 s with 1 min intervals, at 22 kHz) using the Ultrasonic Disintegrator UD-20 (Techpan, Warsaw, Poland). The cell debris was removed by centrifugation at 14,000× *g* and 4 °C for 15 min. The resulting supernatant, a cell-free extract, was used as a crude enzyme preparation to determine the activity of enzymes. The protein content in the extracts was determined according to [53].

The activity of enzymes associated with PAH degradation was assayed spectrophotometrically by using an Evolution 60 spectrometer (Thermo Scientific, USA). NADH-phenanthrenequinone reductase (EC 1.6.5.5; PQR) activity was measured by monitoring the oxidation of NADH at 340 nm according to [54]. The activity of protocatechuate 2,3-dioxygenase (EC 1.13.11.x; 2,3-PCD) was determined by observing the accumulation of 2-hydroxymuconic semialdehyde at 375 nm [55,56]. Protocatechuate 3,4-dioxygenase (EC 1.13.11.3; 3,4-PCD) activity was determined by monitoring the decrease in absorbance at 290 nm, as described by Iwagami et al. [57]. Protocatechuate 4,5-dioxygenase (EC 1.13.11.8; 4,5-PCD) was monitored according to an increase in optical density at 410 nm [58]. The activities of all enzymes were expressed in units (U), defined as μmol of oxidized substrate per minute per mg of protein. The effect of nickel on the activity of enzymes was assessed by introducing nickel ions (to final concentrations of 0.1–5 mM) into the reaction mixtures and measuring the residual enzymatic activity.

### 2.12. Statistics

All experiments were performed in triplicate. All data obtained were subjected to statistical processing based on a preliminary check of the samples for normality of distribution according to the Kolmogorov–Smirnov criterion, calculating the mean values, for comparison of which the standard deviation and confidence interval indicators were used at *p* ≤ 0.05. Calculations were performed in Microsoft Excel 2007. In addition, comparisons of means were performed using Fisher’s test and least significant difference (*p* ≤ 0.05) in one-way analysis of variance (ANOVA). The STATISTICA 13.0 package (TIBCO Software Inc. 2017, Statsoft, Moscow, Russia) was used for data processing and analysis.

## 3. Results

### 3.1. Taxonomic Characteristics of Microorganisms

#### 3.1.1. 16S rRNA Gene Sequence Analysis

The 16S rRNA gene sequences of Rsf11 were obtained both by extraction from whole genome (JBEAAL000000000) and by the conventional PCR amplification and Sanger sequencing (OR826142). As recommended by Chun et al. [59], these sequences were compared to ensure the authenticity of genome data. The comparison showed their 100% similarity.

In 16S rRNA gene sequence analysis for detection of relevant not-yet-genome-sequenced type strains, the input nucleotide matrix comprised 18 operational taxonomic units and 1477 characters, 138 of which were variable and 107 of which were parsimony informative. The base frequency check indicated no compositional bias (*p* = 1.00, α = 0.05). ML analysis under the GTR+GAMMA model yielded a highest log likelihood of -3799.16, whereas the estimated alpha parameter was 0.02. The ML bootstrapping did not converge; hence, 1000 replicates were conducted; the average support was 68.33%. MP analysis yielded a best score of 323 (consistency index 0.56, retention index 0.70) and a single best tree. The MP bootstrapping average support was 84.93%.

**Figure 1:**
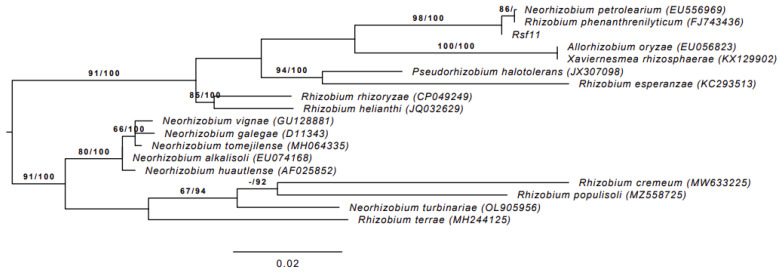
ML phylogenetic tree of strain Rsf11 and closely related type strains inferred from 16S rRNA gene sequences under the GTR+GAMMA model. The tree was rooted at the midpoint. The branches are scaled in terms of the expected number of substitutions per site. The numbers above the branches are support values when larger than 60% from ML (left) and MP (right) bootstrapping. GenBank accession numbers are shown in parentheses. The *Rhizobium terrae* and *Rhizobium populisoli* type strains were assigned to *Neorhizobium* [64] and *Rhizobium phenanthrenilyticum* type strain to *Neorhizobium petrolearium* [23,61].

The phylogenetic relationships of strain Rsf11 with closely related type strains are presented in Figure 1. According to the figure, the strain under study is nested in Rhizobiaceae, clustering with *Neorhizobium petrolearium* and *Rhizobium phenanthrenilyticum* type strains. Moreover, the similarity of Rsf11 with both of these type strains is 99.86%, while with the rest it is less than the species threshold of 98.65% proposed by Kim et al. [60]. It is important to keep in mind that the *R. phenanthrenilyticum* type strain was subsequently re-classified as *N. petrolearium* [23,61].

#### 3.1.2. Whole-Genome-Based Taxonomic Analysis

**Figure 2:**
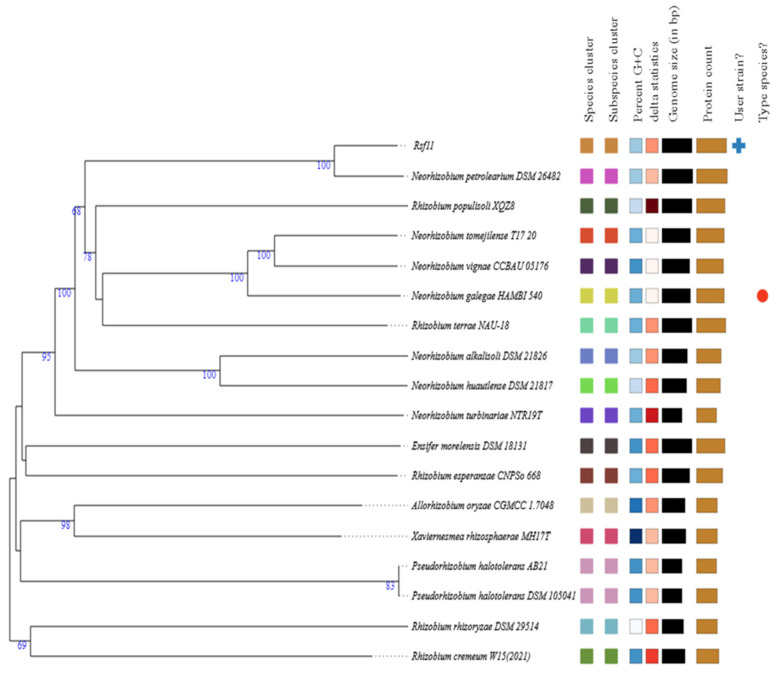
Phylogenetic tree of strain Rsf11 and closely related type strains inferred from GBDP distances calculated from genome sequences. The branch lengths are scaled in terms of GBDP distance formula *d*_5_. The numbers near branches are GBDP pseudo-bootstrap support values > 60% from 100 replications, with an average branch support of 74.4%. The tree was rooted at the midpoint. The *Rhizobium terrae* and *Rhizobium populisoli* type strains were assigned to *Neorhizobium* [64].

The circle of type strains closely related to Rsf11 from the TIGS database included 17 members of the family Rhizobiaceae. Details of these strains are summarized in Table S2. The resulting species and subspecies clusters within the considered circle of bacteria, as well as the phylogenetic relationships between these bacteria, are shown in Figure 2. As can be seen, the clustering yielded seventeen species clusters, and the strain under study was assigned to one of these. Moreover, it was located in one of seventeen subspecies clusters. The Rsf11 strain demonstrated the closest relationship with the type strain of *N. petrolearium* assigned to the other species cluster. Pairwise comparisons of the Rsf11 genome vs. closely related type-strain genomes in terms of dDDH, OrthoANIu, and difference in G+C content are shown in Table 1. As can be concluded from Table 1, the strain under study has the best similarity to the *N. petrolearium* type strain. In particular, the dDDH value calculated using formula *d*_4_, which is independent of genome length and therefore robust to the use of incomplete draft genomes [26,38], is 66.7%, which is below the species threshold of 70% [34]. The OrthoANIu value, 95.92%, is the boundary value for species delineation since the cut-off occurs at 95–96% [62]. The G+C content difference, 0.06%, does not go beyond the species boundary of 1% [63]. The dDDH and OrthoANIu values for the rest type strains are significantly below the 70% and 95–96% thresholds, respectively.

#### 3.1.3. Cultural, Morphological, Physiological, and Biochemical Characteristics

**Figure 3:**
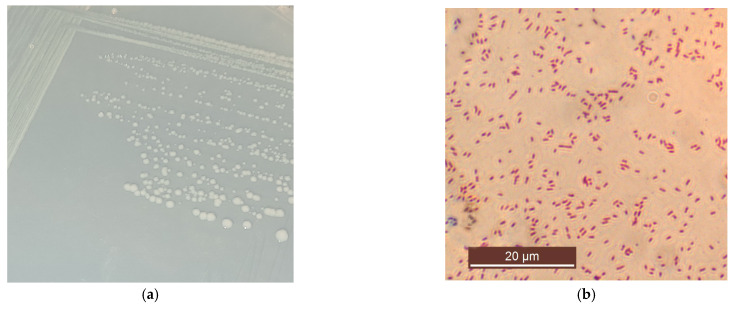

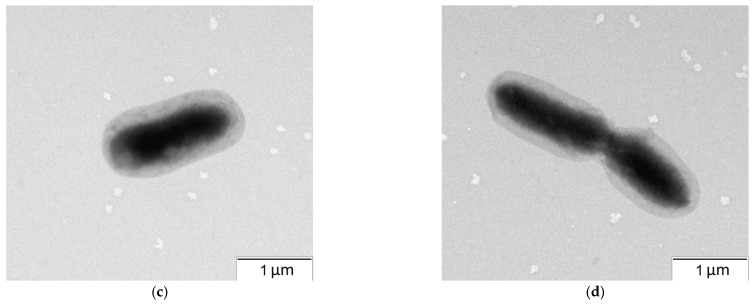
Cultural-morphological characteristics of strain Rsf11: (**a**) 48 h colonies on YMA medium; (**b**) Gram-negative staining of microbial cells; (**c**) transmission electron microscopy of a single cell grown on YMA for 48 h; (**d**) transmission electron microscopy of Rsf11 cell division.

After 2–3 days of incubation at 30 °C, colonies of rhizobacterial strain Rsf11 on YMA medium were small, circular, smooth, convex, white, and translucent; it usually had a diameter of about 2 mm and did not produce a pigment diffusing into agar (Figure 3a). When grown in a liquid medium, it forms a uniform turbidity with the medium. In the presence of 2.5 NaCl, it does not grow; optimum NaCl for growth is 1%. The pH range for growth is 6.0–9.0, and the optimum pH is 7.0–8.0. The range temperature for growth is 10–42 °C, and the optimum temperature is 30 °C.

In 42 h culture grown on YMA, rod-shaped Gram-negative cells (1.5–2.0 µm × 0.6–0.9 µm), located singly, are present (Figure 3b–d). They are mobile, do not form spores or capsules, and accumulate poly-β-hydroxybutyrate.

The culture was oxidase-, catalase-, and urease-positive. It produced nitrate and nitrite reductase but did not produce arginine hydrolase, lysine decarboxylase, phenylalanine deaminase, lipase, lecithinase, or indole. It hydrolyzed esculin but not gelatin or starch. Acid formation was observed in an OF test with glucose, galactose, fructose, rhamnose, xylose, mannose, lactose, arabinose, and mannitol. According to API^®^ 20NE tests, this culture produced β-galactosidase, assimilated D-glucose, L-arabinose, D-mannose, D-mannitol, D-maltose, N-acetylglucosamine, potassium gluconate, and adipic acid but did not assimilate capric, malic, phenylacetic acids, or citrate (Table 2).

#### 3.1.4. Differential Characteristics

Characteristics that differentiate strain Rsf11 from the closely related type strains of the *Neorhizobium* species are summarized in Table 2. Compared to *N. petrolearium* DSM 26482, the most closely related type strain, the microorganism under study has a larger cell size, does not grow at 2% NaCl, does not produce phenylalanine deaminase, and does not assimilate maltose, sucrose, or sorbitol. No characteristics unique to the Rsf11 strain were found.

Based on the taxonomic analyzes carried out, a conclusion was made that strain Rsf11 belongs to a new species of the genus *Neorhizobium*, for which the name *N. phenanthreniclasticum* sp. nov. is proposed.

#### 3.1.5. Description of *N. phenanthreniclasticum* sp. nov.

The etymology of the species name “*phenanthreniclasticum*” is as follows: phe.nan.thre.ni.clas’ti.cum. N.L. neut. n. *phenanthrenum*, phenanthrene; Gr. masc. adj. *klastos*, broken in pieces; N.L. neut. adj. *phenanthreniclasticum*, breaking phenanthrene into pieces.

Colonies are circular, smooth, convex, white, and translucent on YMA at 30 °C and have a diameter of about 2 mm. The pH range for growth is 6.0–9.0, and the optimum pH is 7.0–8.0. The temperature range for growth is 10–42 °C, and the optimum temperature is 30 °C. Cells are rod-shaped, Gram-negative, non-endospore-forming, and aerobic (0.8 × 1.9 mm). The culture is oxidase-, catalase- and urease-positive. Nitrate is reduced to nitrogen. Arginine hydrolase, lysine decarboxylase, phenylalanine deaminase, lipase, lecithinase, and indole are not produced. This species hydrolyzes esculin but not gelatin, starch, or Tween 80. Acid formation was observed in an OF test with glucose, galactose, fructose, rhamnose, xylose, mannose, lactose, arabinose, and mannitol, but no fermentation was noted. According to the results of API^®^ 20NE tests, this species produced β-galactosidase and assimilated D-glucose, L-arabinose, D-mannose, D-mannitol, D-maltose, N-acetylglucosamine, potassium gluconate, and adipic acid but did not assimilate capric, malic, phenylacetic acids, or citrate.

The type strain, Rsf11 (=IBPPM 350), was isolated from the hydrocarbon-contaminated rhizosphere of *Medicago sativa* L., Saratov Region, Russia. The genomic DNA G+C content is 60.6 mol%. The GenBank accession for the Rsf11 16S rRNA gene sequence is OR826142. The GenBank accession for the Rsf11 whole-genome shotgun project is JBEAAL000000000 (BioProject ID, PRJNA1117657; BioSample ID, SAMN41578736; Assembly ID, GCA_040066075.1).

### 3.2. Remediating and Plant-Growth-Promoting Potential of Rsf11

After isolation, the strain was tested for remediating and plant-growth-promoting abilities. The results of examination are given in Table 3.

According to the data obtained, the plant-growth-promoting potential of strain Rsf11 may be associated with atmospheric nitrogen fixation, the synthesis of phytohormone indole-3-acetic acid (IAA), and siderophore production. However, the values of nitrogen fixation and IAA production by Rsf11 were too small to characterize this strain as promising PGPR.

At the same time, the strain demonstrated remediation potential associated with the ability to degrade PAHs (phenanthrene, fluorene, and anthracene) and showed resistance to the presence of nickel in the environment. It is these properties of Rsf11 that we studied in more detail in further research.

### 3.3. Phenanthrene Degradation

**Figure 4:**
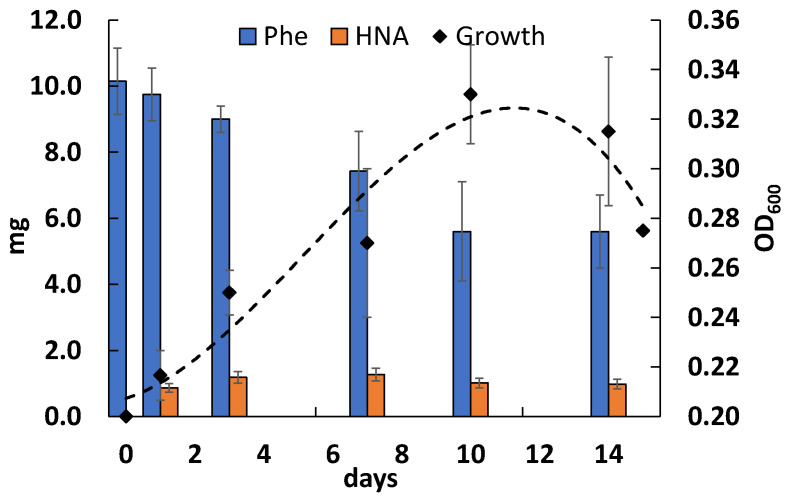
Degradation of phenanthrene as a sole carbon and energy source and HNA formation during the growth of the Rsf11 strain in mineral medium.

**Figure 5:**
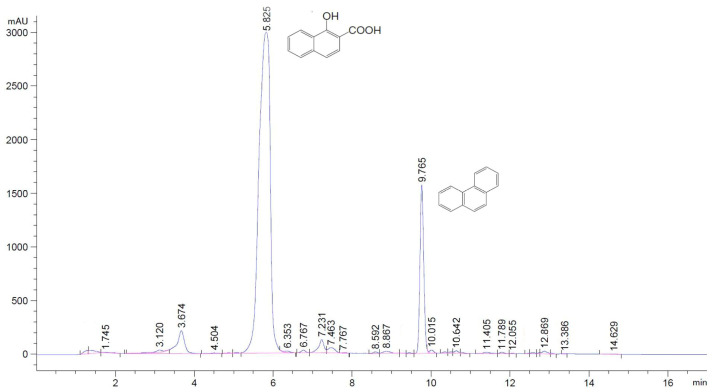
HPLC chromatogram of the ethyl acetate extract of the medium after 7 days’ cultivation of bacterial strain Rsf11 with phenanthrene (0.2 g L^−1^).

A study of the ability of the strain Rsf11 to grow on and to degrade a phenanthrene showed that the growth of the bacterium on a medium with PAH reached a maximum at 10 days (Figure 4). However, the bacterial biomass accumulation was not considerable. On 10th day of cultivation, the degradation of phenanthrene by the microorganism studied reached a maximum value of 58%. The maximum accumulation of 1-hydroxy-2-naphthoic acid (HNA), a predominant phenanthrene catabolite, was observed on the 7th day of cultivation (Figure 4 and Figure 5).

On the chromatograms of ethyl acetate extracts of the culture liquid, two peaks were clearly dominated: The first one with Rt 5.825 coincided with the HNA standard, and the second one with Rt 9.765 corresponded to the phenanthrene standard used (Figure 5).

### 3.4. Nickel Resistance

**Figure 6:**
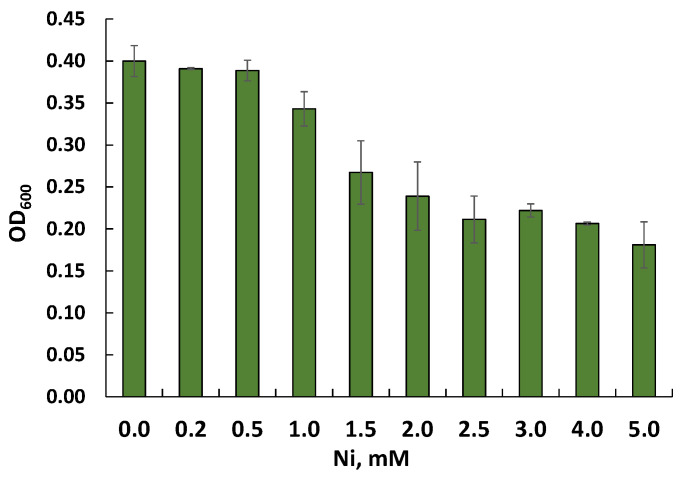
Effect of nickel on growth of the Rsf11 strain in LB medium.

A preliminary study of the resistance of Rsf11 to nickel ions by assessing growth on agar and in liquid LB medium with the addition of nickel ions made it possible to determine the MTC and MIC values for this strain as 1.0 and 1.5 mM, respectively (Figure 6). Additional splitting of the nickel concentration range allowed us to clarify the MTC and MIC values as 1.3 and 1.4 mM.

**Figure 7:**
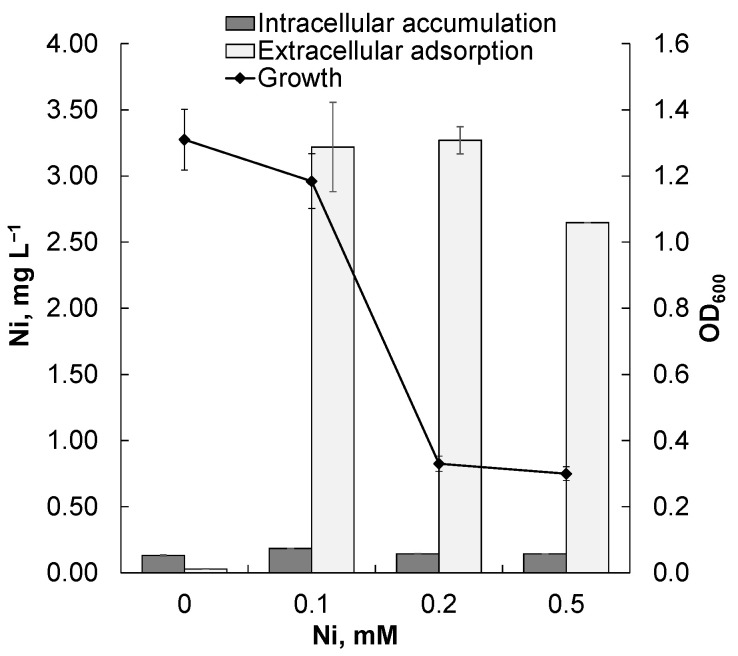
Intracellular accumulation and extracellular adsorption of nickel by Rsf11 cells.

Analysis of the interaction of strain Rsf11 with the nickel ions present in the medium made it possible to characterize the extracellular adsorption and intracellular accumulation of metal by bacterial cells (Figure 7).

From the presented data, it is clear that the extracellular adsorption of nickel is the leading mechanism of resistance of the strain Rsf11 studied to this metal.

### 3.5. Effect of Nickel Ions on Phenanthrene Degradation

**Figure 8:**
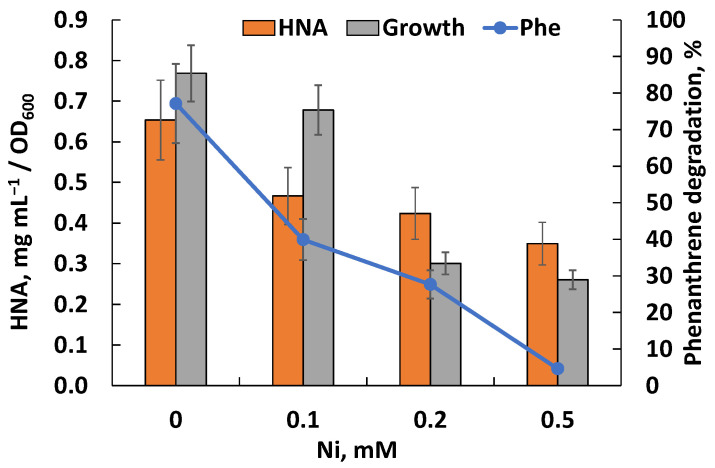
Effect of nickel on growth and phenanthrene degradation by the Rsf11 strain.

The presence of the nickel in the cultivation medium inhibited growth and phenanthrene degradation but did not prevent the formation of HNA as the key intermediate in the Rsf11 strain (Figure 8). At a concentration of 0.1 mM, nickel reduced the degradation of PAH by 23%, and at a concentration of 0.5 mM, by 95%.

**Figure 9:**
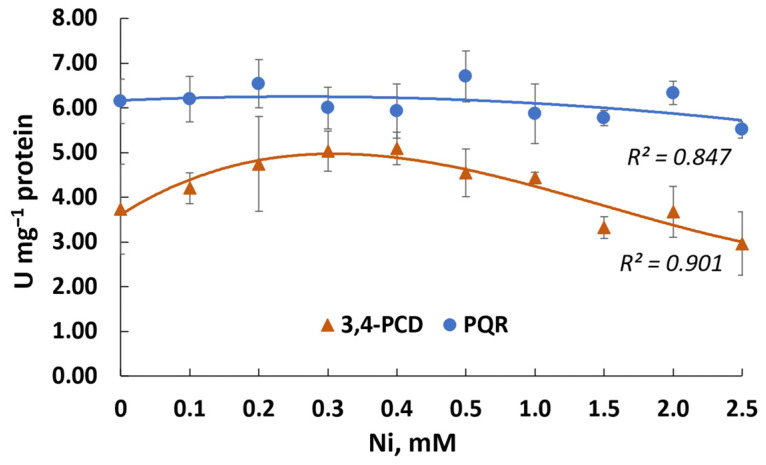
The effect of nickel on the activity of PQR and 3,4-PCD involved in the degradation of phenanthrene by the Rsf11 strain.

The activity of enzymes involved in phenanthrene metabolism, PQR and 3,4-PCD, was detected at a level of 6 and 3.7 μmol min^−1^ mg^−1^ protein, respectively, in the Rsf11 strain (Figure 9).

It was found that under the influence of nickel, the activity of PQR did not change significantly, while the activity of 3,4-PCD even increased by 36.5% in nickel concentrations ranging from 0.1 to 1.0 mM.

### 3.6. Genetic Potential of PAH Transformation and Nickel Resistance

Genes of the Rsf11 strain potentially involved in PAH transformation and nickel resistance are summarized in Table S3 and Table S4, respectively.

According to Table S3, the strain under study carries genes for the naphthalene degradation pathway. In particular, the *nahD* and *nahE* genes encode the enzymes (EC: 5.99.1.4 and EC: 4.1.2.45, respectively) of the upper naphthalene degradation pathway, while the *adh* genes encode an enzyme (EC: 1.1.1.1) of the upper methylnaphthalene degradation pathway. The genes *catE*, *gtdA*, *nahO* (*bphJ*, *xylQ*, *tesF*), *nahM* (*bphI*, *xylK*, *tesG*), and the *sdgC*-like gene are responsible for the synthesis of enzymes (EC: 1.13.11.2, 1.13.11.4, 1.2.1.10/1.2.1.87, 4.1.3.39/4.1.3.43, and 1.14.13.209, respectively) from the lower naphthalene degradation pathway. One of the phthalate degradation pathway genes, *pht5*, the protein product of which (EC: 4.1.1.55) catalyzes the conversion of 4,5-dihydroxyphthalate to protocatechuate, has been revealed in the Rsf11 strain. Genes for the protocatechuate branch of the β-ketoadipate pathway were identified and organized into two operons: *pcaQ-pcaDCHGB* and *pcaR-pcaIJF*. The protocatechuate *meta*-cleavage pathway genes *ligA*, *ligB*, *ligI*, *galB*, *galC* (*ligK*), and *galD* were also discovered. In addition, the cytochrome P450 (*bioI*, *cyp105A1*, *pksS*) and glutathione-S-transferase (*gst*) genes, related to the so-called xenobiotic detoxification genes, were found.

According to Table S4, the Rsf11 strain has genes for the biosynthesis and subsequent transport to the cell surface of capsular polysaccharides (*kps*), exopolysaccharides (*exo*, etc.), and lipopolysaccharides (*lpt*, *lpx*, *kds* etc.). It also possesses nickel-export genes such as *dmeF* and *fieF*, which encode CDF family transporters; *rcnA*, which encodes NicO family transporter; and *atm1*, which encodes ATP-binding cassette transporter. Finally, the genes responsible for the production of cytoplasmic (*groES*, *hspA*, *slyD*, and *ureE* genes) and periplasmic (*nikA*-like gene) nickel-binding proteins were identified in the studied strain.

## 4. Discussion

The diversity of PAH-degrading microorganisms is great enough and has been repeatedly cited in reviews [77,78,79,80]. Among the wide range of PAH-degraders, in our opinion, rhizobia, as nitrogen-fixing microorganisms that live in soil and are capable of forming close symbiosis with plants, deserve special attention. The ability to fertilize the soil with mineral nitrogen and stimulate plant growth are important advantages of rhizobia over other pollutant-degrading microorganisms, especially for phytoremediation, which is increasingly used in biotechnology to clean up soil from oil pollution [20]. A number of studies have shown the significant enrichment of soil contaminated with petroleum hydrocarbons or PAHs by rhizobia of various genera (such as *Cupriavidus*, *Allorhizobium*, *Neorhizobium*, *Pararhizobium*, and *Rhizobium* [81,82]). These observations are supported by the detection of remediating activity against organic pollutants, including PAHs, in members of this group of symbiotic bacteria [24,83,84].

The degradation of phenanthrene by rhizobia has been characterized in most detail using the example of the strain *Sinorhizobium* sp. C4 [22,85]. Sixteen metabolites of phenanthrene were identified, and a metabolic map was proposed that characterizes the pathways of phenanthrene degradation by this symbiotic microorganism. In addition, comparative metabolic reactions of the *Sinorhizobium* sp. C4 during phenanthrene degradation by this strain were studied [85]. In particular, changes affecting the Krebs cycle (TCA cycle), pyruvate metabolism, biosynthesis of cofactor, fatty acid composition, and polyhydroxyalkanoate biosynthesis were found during PAH degradation by C4. The accumulation of sulfur-containing amino acids and niacin indicated possible oxidative stress conditions during phenanthrene metabolism by this bacterium [85]. The ability of *Rhizobium tropici* CIAT899 to grow in the presence of phenanthrene (40 μg/mL) and benzo[a]pyrene (60 μg/mL) was also reported [83]. The authors observed the loss of PAHs in the medium using GC–MS but did not identify any metabolites.

In 2011, Wen et al. presented a bacterial strain, F11T, isolated from an oil sludge treatment system and capable of phenanthrene degradation. Based on phylogenetic analysis, as well as biochemical and physiological characteristics, strain F11T was identified as a new species of the genus *Rhizobium*, which was given the name *Rhizobium phenanthrenilyticum* sp. nov. [86]. In 2012, Zhang et al. presented a new bacterial strain, SL-1, isolated from oil-contaminated soil, which, based on phylogenetic analysis, as well as biochemical and physiological characteristics, together with the previously described *Rhizobium phenanthrenilyticum* F11T, were classified and validated as a new species of *Rhizobium petrolearium* [23]. Isolated *Rhizobium petrolearium* strains have been described as degrading phenanthrene [86] as well as various petroleum products such as crude oil, kerosene, diesel, and gasoline [87]. Huang et al. described in detail the degradation of phenanthrene by the *Rhizobium petrolearium* SL-1 strain [84]. Using HPLC and gas chromatography–mass spectrometry (GC-MS), bacterial metabolites of phenanthrene were identified, and it was shown that phenanthrene (100 mg L^−1^) was completely degraded by strain SL-1 at 35 °C, salinity 0.02%, and pH 9.0 after 3 days. It is also mentioned that the strain can also degrade a wide range of polycyclic aromatic hydrocarbons, including naphthalene, fluorene, anthracene, and pyrene. In 2022, the species *Rhizobium petrolearium* was reclassified and assigned to the genus *Neorhizobium*, receiving the name *Neorhizobium petrolearium* [61].

This study reports another PAH-degrading member of the *Neorhizobium* genus, which, according to taxonomic analyses, belongs to a new species of the above genus and for which the name *N. phenanthreniclasticum* sp. nov. is proposed. As well as the rhizobial strains described above, the *N. phenanthreniclasticum* strain Rsf11 studied in this work was isolated as a phenanthrene-degrader from oil-contaminated soil surrounding the roots of the *Medicago sativa* L. plant. Comparing the data obtained by previously reported studies, it can be noted that the activity of the microorganism studied fits into a number of previously characterized phenanthrene-degraders both in terms of the intensity of PAH degradation and the metabolites formed. The data obtained in this work are consistent with the previously described characterization of phenanthrene degradation by the Rsf11 strain [88]. The utilization of phenanthrene by the strain occurred relatively slowly with the formation of HNA, which, in turn, was also capable of transformation (Figure 4). Under periodic cultivation conditions, over 10–14 days, there was probably a depletion of macro- and microelements important for the growth and functioning of the microorganism, which limited the bacterial growth and further phenanthrene biodegradation. The introduction of an additional carbon source, sodium glutamate, led to both increased growth and increased degradation of phenanthrene (Figure 8), thereby confirming the above assumption about the lack of nutrient resources (glutamate is a source of not only carbon but also nitrogen).

In addition to previously reported information on PAH-degrading rhizobia, we have proposed the enzymatic activity of rhizobia in connection with PAH degradation. In the course of our study, we determined the activity of two enzymes, PQR and 3,4-PCD, which may be involved in the PAH catabolism. PQR may be considered as an enzyme of the upper pathways of PAH degradation. The role of this enzyme in PAH degradation is the detoxification of PAH *o*-quinones (formed as a result of autoxidizing PAH dihydrodiols) that prevents loss of PAH catechols from the PAH degradation [89]. Previously, this enzyme was characterized only for *Mycobacterium* sp. [89]. Research into rhizobial PQR should be continued. The activity of 3,4-PCD as an enzyme of the lower pathways of PAH degradation was also revealed in *N. phenanthreniclasticum* strain Rsf11 (Figure 9). This dioxygenase catalyzes the *ortho*-cleavage (3,4-dioxygenation) pathway of protocatechuate degradation.

The list of identified genes involved in the PAH transformation in *N. phenanthreniclasticum* Rsf11 (Table S3) suggests two main options for the utilization of phenanthrene and HNA as its key catabolite to intermediates of the TCA cycle by this microorganism. One option uses the upper naphthalene degradation pathway genes *nahD* and *nahE*, the products of which sequentially convert 2-hydroxychromene-2-carboxylate formed from 1,2-dihydroxynaphthoate into *trans*-o-hydroxybenzylidenepyruvate and then into salicylaldehyde, as well as the lower naphthalene degradation pathway genes *sdgC*-like, *gtdA*, *catE*, *nahO*, and *nahM*, the products of which ensure the conversion of salicylate into the TCA-cycle intermediates either via gentisate or catechol [90]. In addition, *gst* genes encoding glutathione-S-transferase may be involved in the naphthalene degradation pathway. It is known that this enzyme catalyzes the *cis*–*trans* isomerization between 2-hydroxychromene-2-carboxylic acid and *trans*-o-hydroxybenzylidene pyruvic acid due to its covalent attachment of 2-hydroxychromene-2-carboxylic acid [91]. Another option uses the genes of the phthalate degradation pathway (*pht5*), and then the protocatechuate *ortho*-cleavage (*pcaQ*-*pcaDCHGB* and *pcaR*-*pcaIJF*) and/or *meta*-cleavage (*ligA*, *ligB*, *ligI*, *galB*, *galC*, and *galD*) pathways [92].

Nickel is an essential element for living organisms; it is part of a number of metalloenzymes, such as hydroxylases, hydrogenases, urease, and Ni-superoxide dismutase [93,94]. However, in high concentrations, nickel is toxic and dangerous to living organisms, including plants [94,95]. The targeted use of HM (nickel)-resistant plant-growth-promoting rhizobacteria can effectively protect plants from nickel stress and also serve to remediate contaminated soil. In this regard, it seemed important to evaluate the ability of the rhizobia we isolated to exhibit resistance to HMs and, in particular, to nickel.

To date, resistance to nickel has been described for many genera of rhizobia: *Rhizobium*, from 4 mg L^−1^ [96] to 1000 mg L^−1^ [97]; *Bradyrhizobium,* from 10 mg L^−1^ [98] up to 880.5 mg L^−1^ (15 mM, [99]); *Ensifer*, 8–12 mg L^−1^ [96]; *Sinorhizobium*, from 10 mg L^−1^ [98] up to 176 mg L^−1^ (3 mM, [100]); and *Cupriavidus*, up to 1000 mg L^−1^ [101]. We did not find any data on nickel resistance among members of the *Neorhizobium* genus, but it has been reported that the *Neorhizobium huautlense* strain T1-17 can be described as resistant to other HMs, such as cadmium (2.2 mM), copper (7.9 mM), and zinc (7.7 mM) [102].

Taking into account the testing conditions, the nickel resistance of the *N. phenanthreniclasticum* Rsf11 strain studied in this research is generally comparable with other rhizobia described but is inferior to some exceptional isolates that demonstrate tolerance to nickel at concentrations of up to 1000 mg L^−1^—*Rhizobium* sp. HGR-4 [97] and representatives of *Cupriavidus* genus (*Cupriavidus paucula*, [101]; *Cupriavidus taiwanensis*, [103]) highly adapted to high concentrations of HMs in the environment, like *Cupriavidus metallidurans* [104].

According to the data obtained (Figure 7), the nickel resistance of *N. phenanthreniclasticum* Rsf11 was primarily due to the extracellular adsorption of this metal by the extracellular polymeric substances (EPSs). This fact indicates the dominance of the extracellular barrier mechanism of its nickel ion resistance over others in this strain, which is consistent with the presence of genes that control the biosynthesis and transport to the cell surface of various polymers (Table S4): capsular polysaccharides (*kps*), as well as exo- (*exo* and etc.) and lipopolysaccharides (*lpt*, *lpx*, *kds*, and etc.). Rhizobia, including members of *Neorhizobium*, are well-known EPS producers, capable of releasing large amounts of polysaccharides into the rhizosphere as well as when grown in pure cultures [105,106]. Moreover, it has been shown that nickel ions preferentially bind to extracellular polymeric substances of a polysaccharide nature [107].

As for other mechanisms of resistance to nickel ions possibly used by the strain studied, these were periplasmic and cytoplasmic sequestration, as well as efflux outside the cell (Table S4). The first can be mediated by the NikA-like substrate-binding protein, which is a component of the strictly controlled peptide/nickel transport system [108]. The second can occur through the chaperone SlyD, the chaperonin GroES, the heat shock protein HspA, and the nickel accessory protein UreE [108]. The third can be implemented by using the ABC heavy metal exporter Atm1 [109], the CDF family exporters DmeF and FieF [110], and the NicO family exporter RcnA [111].

A distinct inhibition of the growth of *N. phenanthreniclasticum* Rsf11 at a nickel concentration of >0.1 mM accompanied by a decrease in the phenanthrene degradation and the HNA accumulation was observed, which indicated that catabolic enzymes may be not susceptible or were slightly affected by the HM ions. The data on the enzymatic analysis confirmed this conclusion, demonstrating the absence of the significant inhibition of the activity of the enzymes studied by nickel at concentrations of up to 2.5 mM. Based on the above, we can conclude that the inhibitory effect of nickel on the biodegradation of phenanthrene by the *N. phenanthreniclasticum* strain Rsf11 was associated, first of all, with the suppression of the growth of the microorganism but not with the suppression of the activity of enzymes involved in the degradation of PAHs. However, additional experiments are required to reach a final conclusion. In general, the data obtained here may indicate the prospects of using the strain studied under conditions of mixed contamination with PAHs and HMs.

## 5. Conclusions

The PAH-degrading and nickel-resistant properties of rhizobial strain Rsf11 isolated from the rhizosphere of *Medicago sativa* L. have been investigated in this study. Species identification based on the taxonogenomics approach made it possible to classify the microorganism as a new species of the genus *Neorhizobium*, and the name *Neorhizobium phenanthreniclasticum* sp. nov. was proposed. It was shown that *N. phenanthreniclasticum* Rsf11 is able to destroy three-ringed PAHs such as phenanthrene, fluorene, and anthracene. HNA was revealed as the key metabolite of phenanthrene degradation by Rsf11. The activity of PQR and 3,4-PCD enzymes apparently involved in the degradation of PAHs was determined. Genes potentially involved in PAH catabolism discovered in *N. phenanthreniclasticum* Rsf11 indicate the possibility of phenanthrene transformation both through the naphthalene degradation pathway and through the phthalate and then protocatechuate (*ortho*- and/or *meta*-cleavage) pathways. In addition, the strain studied exhibits pronounced resistance to nickel, which was mainly associated with the extracellular adsorption of metal by EPS. According to the genomic data obtained, in addition to the extracellular barrier mechanism of resistance to nickel ions, Rsf11 has efflux and sequestration (periplasmic and cytoplasmic) mechanisms. The combined presence of phenanthrene and nickel in the medium reduced the degradation of PAH by the microorganism, apparently due to the inhibition of growth processes but not due to inhibition of the activity of the PQR and 3,4-PCD enzymes. Thus, the *N. phenanthreniclasticum* Rsf11 strain can be considered as a promising candidate for use in ecobiotechnology for cleaning technogenically polluted ecosystems.

## Figures and Tables

**Table 1:** Pairwise comparisons of the Rsf11 genome vs. closely related type-strain genomes.

Type Strain	dDDH (%) ^1^						OrthoANIu(%)	G+C Content Difference (%)
	*d*_0_	C.I. *d*_0_	*d*_4_	C.I. *d*_4_	*d*_6_	C.I. *d*_6_		
*Neorhizobium petrolearium* DSM 26482	78.2	[74.2–81.7]	66.7	[63.7–69.5]	78.8	[75.3–81.8]	95.92	0.06
*Neorhizobium vignae* CCBAU 05176	33.2	[29.8–36.7]	24.7	[22.4–27.2]	30.1	[27.2–33.2]	82.01	1.02
*Neorhizobium galegae* HAMBI 540	32.6	[29.2–36.2]	24.7	[22.4–27.2]	29.6	[26.7–32.8]	81.72	0.62
*Rhizobium terrae* NAU-18	34.1	[30.7–37.6]	24.7	[22.4–27.2]	30.7	[27.8–33.8]	81.85	0.84
*Neorhizobium tomejilense* T17 20	32.8	[29.4–36.3]	24.7	[22.4–27.2]	29.8	[26.9–32.9]	81.80	0.85
*Neorhizobium alkalisoli* DSM 21826	30.5	[27.1–34.1]	23.7	[21.4–26.2]	27.8	[24.9–30.9]	81.20	0.3
*Neorhizobium huautlense* DSM 21817	30.1	[26.7–33.7]	23.5	[21.2–26.0]	27.5	[24.6–30.6]	80.63	0.57
*Rhizobium populisoli* XQZ8	27.7	[24.3–31.3]	23.0	[20.7–25.5]	25.6	[22.7–28.7]	80.33	0.54
*Xaviernesmea rhizosphaerae* MH17T	14.4	[11.6–17.8]	21.8	[19.5–24.2]	14.7	[12.2–17.5]	74.85	3.9
*Neorhizobium turbinariae* NTR19T	23.3	[20.0–26.9]	21.6	[19.4–24.1]	22.0	[19.2–25.0]	78.85	0.62
*Rhizobium cremeum* W15(2021)	16.2	[13.2–19.7]	21.2	[19.0–23.7]	16.2	[13.6–19.1]	76.03	1.04
*Allorhizobium oryzae* CGMCC 1.7048	14.4	[11.5–17.8]	20.6	[18.4–23.1]	14.6	[12.1–17.4]	74.21	2.18
*Rhizobium esperanzae* CNPSo 668	16.3	[13.3–19.8]	20.6	[18.4–23.0]	16.2	[13.7–19.2]	75.91	0.45
*Ensifer morelensis* DSM 18131	15.0	[12.1–18.4]	20.4	[18.2–22.8]	15.1	[12.6–18.0]	74.64	1.25
*Pseudorhizobium halotolerans* DSM 105041	18.1	[15.0–21.7]	20.3	[18.1–22.7]	17.7	[15.1–20.7]	nd	1.09
*Pseudorhizobium halotolerans* AB21	18.1	[15.0–21.7]	20.3	[18.1–22.7]	17.7	[15.1–20.7]	76.65	1.08
*Rhizobium rhizoryzae* DSM 29514	14.9	[12.0–18.3]	20.1	[17.9–22.5]	15.0	[12.6–17.9]	74.35	2.64

^1^ The dDDH values are provided along with their confidence intervals (C.I.) for the three different GBDP formulas [26,38]: Formula *d*_0_ (also known as GGDC formula 1): length of all HSPs divided by total genome length; formula *d*_4_ (also known as GGDC formula 2): sum of all identities found in HSPs divided by overall HSP length (*d*_4_ is independent of genome length and is thus robust against the use of incomplete draft genomes); formula *d*_6_ (also known as GGDC formula 3): sum of all identities found in HSPs divided by total genome length.

**Table 2:** Differential characteristics of Rsf11 and the closely related type strains of *Neorhizobium* species.

Characteristic	1	2	3	4	5	6	7	8	9
Cell size, µm	1.9 × 0.8	1.75 × 0.75	1.25 × 0.6		1.45 × 0.65		2.0 × 0.4		
Halotolerance:									
2% NaCl	−	+	+	−			+	+	
3% NaCl	−				−		+		
Temperatures:									
37 °C	+	+	+	−		−	+		
42 °C	+			−	+		+	−	
Production:									
phenylalanine deaminase	−	+					+		
catalase	+	+	+	−		+	+		
oxidase	+	+	+	+	−	+	+		
urease	+	+	+	+	−		+		
nitrate reductase	+	+	+	+	+		−	+	−
nitrite reductase	+			−				+	
indole	−	−	−	−	−		+	−	−
Hydrolysis:									
arginine	−		−				+		−
starch	−							−	
esculin	+	+	+	+				−	
Assimilation:									
maltose	−	+				+		−	
rhamnose	+	+	+				+	−	
sucrose	−	+	+				+	−	−
fructose	+		+		−	+	+	+	
mannitol	+	+	+			+		−	
inositol	−					−	+		
sorbitol	−	+	+	−		+	+	−	−
N-acetylglucosamine	+	+						−	−
malate	−	−	+				+		
G+C content (%)	60.6	60.5	61.6	61.2	61.4	61.4	60.3	60.0	60.1

Notes: 1—Rsf11 strain; 2—Neorhizobium petrolearium DSM 26482 [23,61]; 3—Neorhizobium vignae DSM 25378 [65,66]; 4—Neorhizobium galegae DSM 11542 [67,68]; 5—Neorhizobium terrae NAU 18 [64,69]; 6—Neorhizobium tomejilense T17 20 [70]; 7—Neorhizobium alkalisoli DSM 21826 [68,71]; 8—Neorhizobium huautlense SO_2_ [68,72]; 9—Neorhizobium populisoli XQZ8 [64,73].

**Table 3:** Remediating and plant-growth-promoting potential of the *Rsf11 strain*.

PGPR Potential		Remediation Potential	
Nitrogen fixation	0.08 ± 0.01 nmol C_2_H_4_ h^−1^ mL^−1^	PAH degradation ^1^	Phe, Ant, Flu
P-solubilization	−	Synthetic surfactant degradation ^2^	−
Siderophore production	+	Oil degradation	−
IAA production	3.3 ± 0.3 μg mL^−1^	Heavy-metal resistance ^3^	Ni^2+^ (1.5 mM)

^1^ Kiyohara’s plate test was used to reveal PAH-degrading ability [74]; naphthalene (Nah), phenanthrene (Phe), fluorene (Flu), anthracene (Ant), fluoranthene (Flt), and pyrene (Pyr) were tested. ^2^ Plate test with Dragendorff’s reagent [75] was used to reveal synthetic-surfactant-degrading ability according to [76]. Ethoxylated alcohols (Sintanol DS-10, Sulphonol), ethoxylated alkylphenols (Triton X-100; Neonol APh9–12), and ethoxylated fatty acid (Stearox) were used as substrates. ^3^ HM resistance was tested by plating the bacteria on LB agar medium containing ions of different metals at concentrations ranging from 0 to 5 mM. For the analysis, soluble salts of CuSO_4_, ZnSO_4_, NiSO_4_, CdCl_2_, Pb(NO_3_)_2_, and Na_3_AsO_4_ were used.

## Data Availability

The whole-genome shotgun sequencing project and the 16S rRNA gene sequence presented in this study are openly available in GenBank under the accession numbers JBEAAL000000000 and OR826142, respectively.

## Supplementary Materials

The following supporting information can be downloaded at: https://www.mdpi.com/article/10.3390/microorganisms12081586/s1, Table S1. Features of the Rsf11 genome assembly (GCA_040066075.1); Table S2. TIGS type strains closely related to strain Rsf11 (accessed on 9 June 2024); Table S3. Rsf11 genes potentially involved in transformation of PAHs; Table S4. Rsf11 genes potentially involved in nickel resistance. References [23,61,65,66,67,68,69,70,71,72,73,112,113,114,115,116,117,118,119,120] are cited in the Supplementary Materials.
