# Supplementary material for: Phenanthrene-Degrading and Nickel-Resistant Neorhizobium Strain Isolated from Hydrocarbon-Contaminated Rhizosphere of Medicago sativa L
Source: Microorganisms. 2024 Aug 4;12(8):1586. doi: 10.3390/microorganisms12081586 (PMC11356111; doi:10.3390/microorganisms12081586)
Supplement: Supplementary file 1 [file microorganisms-12-01586-s001.zip › microorganisms-3118735-supplementary.pdf]

**Table S1.** Features of the Rsf11 genome assembly (GCA_040066075.1).

| **Feature** | **Value** |
|---|---|
| *Assembly statistics:* | |
| Genome size (bp) | 6,949,400 |
| Contigs (>500 bp) | 101 |
| Contig N50 (kb) | 179.8 |
| Contig L50 | 11 |
| G+C (%) | 60.6 |
| Genome coverage | 45.7x |
| Assembly level | Contig |
| *Annotation details:* | |
| Genes (total) | 6,781 |
| CDSs (total) | 6,726 |
| Genes (coding) | 6,581 |
| CDSs (with protein) | 6,581 |
| Pseudo Genes | 145 |
| Genes (RNA) | 55 |
| rRNAs (16S, 23S) | 2 (1, 1) |
| complete rRNAs (16S, 23S) | 2 (1, 1) |
| tRNAs | 49 |
| ncRNAs | 4 |

**Table S2.** TIGS type-strains closely related to strain Rsf11 (accessed on 2024.06.09).

| Strain | TYGS ID | Authority | Other deposits | Synonyms | Base pairs | G+C, % | No. proteins | Goldstamp | Bioproject ID | Biosample ID | Assembly ID |
|---|---|---|---|---|---|---|---|---|---|---|---|
| *Neorhizobium petrolearium* DSM 26482 | 126537 | (Zhang et al. 2012) Kuzmanović et al. 2022 | ACCC 11238; KCTC 23288; DSM 21304; SL-1 | *Neorhizobium petrolearium; Rhizobium petrolearium* | 6,969,123 | 60.55 | 6827 | Gp0505702 | | | |
| *Neorhizobium vignae* CCBAU 05176 | 4319 | (Ren et al. 2011) Hördt et al. 2020 | LMG 25447; DSM 25378; HAMBI 3039 | *Neorhizobium vignae; Rhizobium vignae* | 6,343,049 | 61.63 | 6071 | Gp0095595 | PRJNA251791 | SAMN02840656 | GCA_000732195 |
| *Neorhizobium galegae* HAMBI 540 | 3816 | (Lindström 1989) Mousavi et al. 2014 emend. Hördt et al. 2020 | LMG 6214; CIP 105585; ATCC 43677; DSM 11542; JCM 20973; IFO 14965; NBRC 14965 | *Neorhizobium galegae; Rhizobium galegae* | 6,455,027 | 61.23 | 6170 | Gp0081455 | PRJEB1950 | SAMEA3139073 | GCA_000731315 |
| *Rhizobium terrae* NAU-18 | 12205 | Ruan et al. 2020 | KCTC 62418; CCTCC AB 2018075 | *Neorhizobium terrae; Rhizobium terrae* | 6,960,812 | 61.45 | 6561 | | PRJNA474074 | SAMN09291313 | GCA_003425685 |
| *Neorhizobium tomejilense* T17 20 | 70216 | Soenens et al. 2019 | LMG 30623; CECT 9621 | *Neorhizobium tomejilense* | 6,545,966 | 61.46 | 6300 | | PRJNA434362 | SAMN08554955 | GCA_002968845 |
| *Neorhizobium alkalisoli* DSM 21826 | 18998 | (Lu et al. 2009) Mousavi et al. 2014 emend. Hördt et al. 2020 | LMG 24763; CCBAU 1393; HAMBI 3051 | *Neorhizobium alkalisoli; Rhizobium alkalisoli* | 5,869,725 | 60.31 | 5631 | Gp0377375 | PRJNA434371 | SAMN08554967 | GCA_002968635 |
| *Neorhizobium huautlense* DSM 21817 | 11282 | (Wang et al. 1998) Mousavi et al. 2014 emend. Hördt et al. 2020 | LMG 18254; CIP 105757; ATCC BAA-115; HAMBI 2409; SO2; SO2 (The type strain has been deposited in the USDA ARS National Rhizobium Resource Center collection, | *Neorhizobium huautlense; Rhizobium huautlense* | 5,725,939 | 60.04 | 5459 | Gp0377371 | PRJNA434370 | SAMN08554966 | GCA_002968575 |

| Strain | TYGS ID | Authority | Other deposits | Synonyms | Base pairs | G+C, % | No. proteins | Goldstamp | Bioproject ID | Biosample ID | Assembly ID |
|---|---|---|---|---|---|---|---|---|---|---|---|
| | | | USA, and in the Culture Collection of Centro de Investigación sobre Fijación de Nitrógeno at UNAM, Cuernavaca, Mexico. Accession numbers not given.) | | | | | | | | |
| *Rhizobium populisoli* XQZ8 | 70297 | Shen et al. 2022 | JCM 34442; GDMCC 1.2201 | *Neorhizobium populisoli; Rhizobium populisoli* | 6,837,762 | 60.07 | 6419 | | PRJNA746549 | SAMN20209572 | GCA_019430945 |
| *Neorhizobium turbinariae* NTR19T | 115582 | Sun et al. 2023 | MCCC 1K07226; JCM 35342 | *Neorhizobium turbinariae* | 4,656,007 | 61.23 | 4476 | | PRJNA224116 | SAMN27756077 | GCF_023223505 |
| *Xaviernesmea rhizosphaerae* MH17T | 12276 | Kuzmanović et al. 2022 | ACCC 19963; KCTC 52414 | *Rhizobium rhizosphaerae; Xaviernesmea rhizosphaerae* | 5,529,478 | 64.51 | 4698 | Gp0323575 | PRJNA344723 | SAMN05832038 | GCA_001938945 |
| *Rhizobium cremeum* W15(2021) | 147570 | Yang et al. 2022 | CGMCC 1.18731; KACC 22344; W15 | *Rhizobium cremeum* | 5,328,908 | 61.66 | 5113 | | PRJNA224116 | SAMN18012446 | GCF_022884065 |
| *Allorhizobium oryzae* CGMCC 1.7048 | 7842 | (Peng et al. 2008) Mousavi et al. 2015 emend. Hördt et al. 2020 | 505; Alt 505; LMG 24253; HAMBI 3197 | *Allorhizobium oryzae; Rhizobium oryzae; Xaviernesmea oryzae* | 5,387,643 | 62.79 | 4776 | Gp0112589 | PRJEB16857 | SAMN04487976 | GCA_900109605 |
| *Rhizobium esperanzae* CNPSo 668 | 11222 | Cordeiro et al. 2017 emend. Hördt et al. 2020 | 668; LMG 30030; U 10001; UMR 1320; Z87-8 | *Rhizobium esperanzae* | 6,294,057 | 61.06 | 5834 | Gp0387104 | PRJNA378648 | SAMN06555453 | GCA_002204185 |
| *Ensifer morelensis* DSM 18131 | 126532 | (Wang et al. 2002) Wang et al. 2015 | LMG 21331; DSM 18131; NBRC 100387; CFN E1007; Lc04 | *Ensifer morelensis; Sinorhizobium morelense* | 6,824,949 | 61.86 | 6407 | Gp0505705 | | | |

| Strain | TYGS ID | Authority | Other deposits | Synonyms | Base pairs | G+C, % | No. proteins | Goldstamp | Bioproject ID | Biosample ID | Assembly ID |
|---|---|---|---|---|---|---|---|---|---|---|---|
| *Pseudorhizobium halotolerans* DSM 105041 | 15712 | Lassalle et al. 2021 | AB21; DSM 105041; JCM 17536; KEMC 224; KEMC 224-056 | *Pseudorhizobium halotolerans; Rhizobium halotolerans* | 4,690,698 | 61.7 | 4642 | Gp0456383 | | | |
| *Pseudorhizobium halotolerans* AB21 | 7583 | Lassalle et al. 2021 | AB21; DSM 105041; JCM 17536; KEMC 224; KEMC 224-056 | *Pseudorhizobium halotolerans; Rhizobium halotolerans* | 4,699,964 | 61.69 | 4584 | | PRJEB21840 | SAMEA5739409 | GCA_902153235 |
| *Rhizobium rhizoryzae* DSM 29514 | 7637 | Zhang et al. 2014 | ACCC 5916; KCTC 23652; DSM 19478; J3-AN59 | *Affinirhizobium rhizoryzae; Rhizobium rhizoryzae* | 5,021,166 | 57.97 | 4746 | Gp0400924 | | | |

**References**


1. Cordeiro AB, Ribeiro RA, Helene LCF, Hungria M. *Rhizobium esperanzae* sp. nov., a N2-fixing root symbiont of *Phaseolus vulgaris* from Mexican soils. Int J Syst Evol Microbiol. 2017 Oct;67(10):3937-3945. doi: 10.1099/ijsem.0.002225.
2. Hördt A, López MG, Meier-Kolthoff JP, Schleuning M, Weinhold LM, Tindall BJ, Gronow S, Kyrpides NC, Woyke T, Göker M. Analysis of 1,000+ Type-Strain Genomes Substantially Improves Taxonomic Classification of Alphaproteobacteria. Front Microbiol. 2020 Apr 7;11:468. doi: 10.3389/fmicb.2020.00468.
3. Wang ET, van Berkum P, Beyene D, Sui XH, Dorado O, Chen WX, Martínez-Romero E. *Rhizobium huautlense* sp. nov., a symbiont of *Sesbania herbacea* that has a close phylogenetic relationship with *Rhizobium galegae*. Int J Syst Bacteriol. 1998 Jul;48 Pt 3:687-99. doi: 10.1099/00207713-48-3-687.
4. Mousavi SA, Österman J, Wahlberg N, Nesme X, Lavire C, Vial L, Paulin L, de Lajudie P, Lindström K. Phylogeny of the *Rhizobium-Allorhizobium-Agrobacterium* clade supports the delineation of *Neorhizobium* gen. nov. Syst Appl Microbiol. 2014 May;37(3):208-15. doi: 10.1016/j.syapm.2013.12.007.
5. Sun H, Miao Z, Liu S, Liu X, Chen B, Liao B, Xiao B. *Neorhizobium turbinariae* sp. nov., a coral-beneficial bacterium isolated from *Turbinaria peltata*. Int J Syst Evol Microbiol. 2023 Sep;73(9). doi: 10.1099/ijsem.0.006057.
6. Ruan ZP, Cao WM, Zhang X, Liu JT, Zhu JC, Hu B, Jiang JD. *Rhizobium terrae* sp. nov., Isolated from an Oil-Contaminated Soil in China. Curr Microbiol. 2020 Jun;77(6):1117-1124. doi: 10.1007/s00284-020-01889-5.
7. Kuzmanović N, Fagorzi C, Mengoni A, Lassalle F, diCenzo GC. Taxonomy of Rhizobiaceae revisited: proposal of a new framework for genus delimitation. Int J Syst Evol Microbiol. 2022 Mar;72(3):005243. doi: 10.1099/ijsem.0.005243.
8. Wang ET, Tan ZY, Willems A, Fernández-López M, Reinhold-Hurek B, Martínez-Romero E. *Sinorhizobium morelense* sp. nov., a Leucaena leucocephala-associated bacterium that is highly resistant to multiple antibiotics. Int J Syst Evol Microbiol. 2002 Sep;52(Pt 5):1687-93. doi: 10.1099/00207713-52-5-1687.
9. Wang YC, Wang F, Hou BC, Wang ET, Chen WF, Sui XH, Chen WX, Li Y, Zhang YB. Proposal of *Ensifer psoraleae* sp. nov., *Ensifer sesbaniae* sp. nov., *Ensifer morelense* comb. nov. and *Ensifer americanum* comb. nov. Syst Appl Microbiol. 2013 Oct;36(7):467-73. doi: 10.1016/j.syapm.2013.05.001.

10. Zhang X, Li B, Wang H, Sui X, Ma X, Hong Q, Jiang R. *Rhizobium petrolearium* sp. nov., isolated from oil-contaminated soil. Int J Syst Evol Microbiol. 2012 Aug;62(Pt 8):1871-1876. doi: 10.1099/ijs.0.026880-0.
11. Yang E, Liu J, Chen D, Wang S, Xu L, Ma K, Zhang X, Sun L, Wang W. *Rhizobium cremeum* sp. nov., isolated from sewage and capable of acquisition of heavy metal and aromatic compounds resistance genes. Syst Appl Microbiol. 2022 May;45(3):126322. doi: 10.1016/j.syapm.2022.126322.
12. Li Lu Y, Chen WF, Li Han L, Wang ET, Chen WX. *Rhizobium alkalisoli* sp. nov., isolated from Caragana intermedia growing in saline-alkaline soils in the north of China. Int J Syst Evol Microbiol. 2009 Dec;59(Pt 12):3006-11. doi: 10.1099/ijs.0.007237-0.
13. Lindstrom, K. "Rhizobium galegae, a new species of legume root nodule bacteria." Int. J. Syst. Bacteriol. (1989) 39:365-367. doi: 10.1099/00207713-39-3-365
14. Ren DW, Chen WF, Sui XH, Wang ET, Chen WX. *Rhizobium vignae* sp. nov., a symbiotic bacterium isolated from multiple legume species. Int J Syst Evol Microbiol. 2011 Mar;61(Pt 3):580-586. doi: 10.1099/ijs.0.023143-0.
15. Soenens A, Gomila M, Imperial J. *Neorhizobium tomejilense* sp. nov., first non-symbiotic Neorhizobium species isolated from a dryland agricultural soil in southern Spain. Syst Appl Microbiol. 2019 Mar;42(2):128-134. doi: 10.1016/j.syapm.2018.09.001.
16. Shen L, Liu JJ, Liu PX, An MM, He XW, Zhao GZ. A non-symbiotic novel species, *Rhizobium populisoli* sp. nov., isolated from rhizosphere soil of *Populus popularis*. Arch Microbiol. 2022;204(1):50. doi: 10.1007/s00203-021-02706-8.
17. Lassalle F, Dastgheib SMM, Zhao FJ, Zhang J, Verbarg S, Frühling A, Brinkmann H, Osborne TH, Sikorski J, Balloux F, Didelot X, Santini JM, Petersen J. Phylogenomics reveals the basis of adaptation of *Pseudorhizobium* species to extreme environments and supports a taxonomic revision of the genus. Syst Appl Microbiol. 2021 Jan;44(1):126165. doi: 10.1016/j.syapm.2020.126165.
18. Zhang XX, Tang X, Sheirdil RA, Sun L, Ma XT. *Rhizobium rhizoryzae* sp. nov., isolated from rice roots. Int J Syst Evol Microbiol. 2014 Apr;64(Pt 4):1373-1377. doi: 10.1099/ijs.0.056325-0.
19. Peng G, Yuan Q, Li H, Zhang W, Tan Z. *Rhizobium oryzae* sp. nov., isolated from the wild rice Oryza alta. Int J Syst Evol Microbiol. 2008 Sep;58(Pt 9):2158-63. doi: 10.1099/ijs.0.65632-0.
20. Mousavi SA, Willems A, Nesme X, de Lajudie P, Lindström K. Revised phylogeny of Rhizobiaceae: proposal of the delineation of *Pararhizobium* gen. nov., and 13 new species combinations. Syst Appl Microbiol. 2015 Mar;38(2):84-90. doi: 10.1016/j.syapm.2014.12.003.

**Table S3.** Rsf11 genes potentially involved in transformation of PAHs.

| **Locus Tag** | **Gene** | **Gene Product** | **KO Number** | **EC Number** | **Metabolic Pathway** |
|---|---|---|---|---|---|
| ABK249_09835 | *adh* | Alcohol dehydrogenase | K00001 | 1.1.1.1 | Upper naphthalene degradation pathway |
| ABK249_13005, ABK249_18655 | *adhP* | Alcohol dehydrogenase AdhP | K13953 | 1.1.1.1 | |
| ABK249_12320 | *adhC, ADH5, frmA* | S-(hydroxymethyl)glutathione dehydrogenase/alcohol dehydrogenase | K00121 | 1.1.1.1, 1.1.1.284 | |
| ABK249_28075 | *nahE* | *Trans*-o-hydroxybenzylidenepyruvate hydratase-aldolase | K14585 | 4.1.2.45 | |
| ABK249_28080 | *nahD* | 2-hydroxychromene-2-carboxylate isomerase | K14584 | 5.99.1.4 | |
| ABK249_21435 | *sdgC*-like | Bifunctional salicylyl-CoA 5-hydroxylase/oxidoreductase | K21684* | 1.14.13.209 | Lower naphthalene degradation pathway |

| | | | | | |
|---|---|---|---|---|---|
| ABK249_22150 | *catE* | Catechol 2,3-dioxygenase | K07104 | 1.13.11.2 | |
| ABK249_28070 | *gtdA* | Gentisate 1,2-dioxygenase | K00450 | 1.13.11.4 | |
| ABK249_28150 | *bphJ, xylQ, nahO, tesF* | Acetaldehyde/propanal dehydrogenase | K18366 | 1.2.1.10, 1.2.1.87 | |
| ABK249_28155 | *bphI, xylK, nahM, tesG* | 4-hydroxy-2-oxovalerate/4-hydroxy-2-oxohexanoate aldolase | K18365 | 4.1.3.39, 4.1.3.43 | |
| ABK249_29470 | *pht5* | 4,5-dihydroxyphthalate decarboxylase | K04102 | 4.1.1.55 | Phthalate degradation pathway |
| ABK249_17465, ABK249_18275, | *pcaR* | IclR family transcriptional regulator, *pca* regulon regulatory protein | K02624 | – | Protocatechuate branch of the β-ketoadipate pathway (*ortho*-cleavage pathway) |
| ABK249_17470 | *pcaI* | 3-oxoadipate CoA-transferase, alpha subunit | K01031 | 2.8.3.6 | |
| ABK249_17475 | *pcaJ* | 3-oxoadipate CoA-transferase, beta subunit | K01032 | 2.8.3.6 | |
| ABK249_17480, ABK249_32735 | *pcaF* | 3-oxoadipyl-CoA thiolase | K07823 | 2.3.1.174 | |
| ABK249_17870, ABK249_28595 | *pcaB* | 3-carboxy-*cis,cis*-muconate cycloisomerase | K01857 | 5.5.1.2 | |
| ABK249_17875 | *pcaG* | Protocatechuate 3,4-dioxygenase, alpha subunit | K00448 | 1.13.11.3 | |
| ABK249_17880 | *pcaH* | Protocatechuate 3,4-dioxygenase, beta subunit | K00449 | 1.13.11.3 | |
| ABK249_17885 | *pcaC* | 4-carboxymuconolactone decarboxylase | K01607 | 4.1.1.44 | |
| ABK249_17890 | *pcaD* | 3-oxoadipate enol-lactonase | K01055 | 3.1.1.24 | |
| ABK249_17895 | *pcaQ* | pca operon transcription factor PcaQ | K02623 | – | |
| ABK249_06260 | *galB* | 4-oxalomesaconate hydratase | K16515 | 4.2.1.83 | Protocatechuate *meta*-cleavage pathway |
| ABK249_06265 | *galC, ligK* | 4-hydroxy-4-methyl-2-oxoglutarate aldolase | K10218 | 4.1.3.17 | |
| ABK249_06270 | *galD* | 4-oxalomesaconate tautomerase | K16514 | 5.3.2.8 | |
| ABK249_25415 | *ligI* | 2-pyrone-4,6-dicarboxylate lactonase | K10221 | 3.1.1.57 | |
| ABK249_18260 | *ligA* | Protocatechuate 4,5-dioxygenase, alpha chain | K04100* | 1.13.11.8 | |
| ABK249_18265 | *ligB* | Protocatechuate 4,5-dioxygenase, beta chain | K04101 | 1.13.11.8 | |
| ABK249_10875, ABK249_24765 | *cyp105A1* | Vitamin D 1,25-hydroxylase | K21164 | 1.14.15.22 | Cytochrome P450-dependent metabolism of xenobiotics |
| ABK249_11190 | *pksS* | Polyketide biosynthesis cytochrome P450 PksS | – | 1.14.-.- | |
| ABK249_28015 | *bioI* | Biotin biosynthesis cytochrome P450 | – | 1.14.14.46 | |
| ABK249_00085, ABK249_00100, ABK249_03600, ABK249_03750, ABK249_06545, ABK249_07105, ABK249_18835, ABK249_19080, ABK249_19325, ABK249_19695, ABK249_20925, ABK249_22345, ABK249_23430, ABK249_28100, ABK249_30115 | *gst* | Glutathione S-transferase | K00799 | 2.5.1.18 | |

*KO identifiers assigned based on the results of a direct search in the KO database

**Table S4.** Rsf11 genes potentially involved in nickel resistance.

| Locus Tag | Gene | Gene Product | KO Number | EC Number | TC Number | HM resistance mechanism |
|---|---|---|---|---|---|---|
| Capsular polysaccharides | | | | | | Extracellular barrier |
| ABK249_00295, ABK249_20810 | *kpsM* | Capsular polysaccharide transport system permease protein | K09688 | – | 3.A.1.101 | |
| ABK249_00300 | *kpsT* | Capsular polysaccharide transport system ATP-binding protein | K09689 | 7.6.2.12 | 3.A.1.101 | |
| ABK249_00305 | *kpsE* | Capsular polysaccharide transport system permease protein | K10107 | – | 8.A.4 | |
| ABK249_00310 | *kpsC, lipA* | Capsular polysaccharide biosynthesis protein | K07266* | – | – | |
| ABK249_32885, ABK249_32890 | *kpsS, lipB* | Capsular polysaccharide export protein | K07265 | – | – | |
| Exopolysaccharides | | | | | | |
| ABK249_05020 | *pssA* | Acidic exopolysaccharide biosynthesis protein PssA | K25902 | – | – | |
| ABK249_23100 | *cysE* | Serine O-acetyltransferase | K00640 | 2.3.1.30 | – | |
| ABK249_25520 | *wcaF* | Putative colanic acid biosynthesis acetyltransferase WcaF | K03818 | 2.3.1.- | – | |
| ABK249_25535 | *wecA, tagO, rfe* | UDP-GlcNAc:undecaprenyl-phosphate/decaprenyl-phosphate GlcNAc-1-phosphate transferase | K02851 | 2.7.8.33, 2.7.8.35 | – | |
| ABK249_28735 | *exoP, vpsO* | Polysaccharide biosynthesis transport protein | K16554 | 2.7.10.3 | 8.A.3.1 | |
| ABK249_28740 | *UGP2, galU, galF* | UTP--glucose-1-phosphate uridylyltransferase | K00963 | 2.7.7.9 | – | |
| ABK249_28745 | *exoO* | Succinoglycan biosynthesis protein ExoO | K16555 | 2.4.-.- | – | |
| ABK249_28750 | *exoM* | Succinoglycan biosynthesis protein ExoM | K16556 | 2.4.-.- | – | |
| ABK249_28755 | *exoA* | Succinoglycan biosynthesis protein ExoA | K16557 | 2.4.-.- | – | |
| ABK249_28760 | *exoL* | Succinoglycan biosynthesis protein ExoL | K16558 | 2.-.-.- | – | |
| ABK249_28765 | *exoK* | Endo-1,3-1,4-beta-glycanase ExoK | K16559 | 3.2.1.- | – | |
| ABK249_28770 | *exoH* | Succinoglycan biosynthesis protein ExoH | K16560 | – | – | |
| ABK249_28775 | *exoW* | Succinoglycan biosynthesis protein ExoW | K16562 | 2.4.-.- | – | |
| ABK249_28780 | *exoV* | Succinoglycan biosynthesis protein ExoV | K16563 | – | – | |
| ABK249_28785 | *exoU* | Succinoglycan biosynthesis protein ExoU | K16564 | 2.4.-.- | – | |
| ABK249_28790 | *exoX, syrA* | Exopolysaccharide production regulatory protein | K16565 | – | – | |
| ABK249_28795 | *exoY* | Exopolysaccharide production protein ExoY | K16566 | – | – | |
| ABK249_28800 | *exoF* | Polysaccharide biosynthesis/export protein ExoF | K16552 | – | 1.B.18.1 | |
| ABK249_28805 | *exoQ* | Exopolysaccharide production protein ExoQ | K16567 | – | – | |
| ABK249_28810 | *exoZ* | Exopolysaccharide production protein ExoZ | K16568 | – | – | |
| Lipopolysaccharides | | | | | | |
| ABK249_01175 | *lpxK* | tetraacyldisaccharide 4'-kinase | K00912 | 2.7.1.130 | – | |
| ABK249_01185 | *kdtA, waaA* | 3-deoxy-D-manno-octulosonic-acid transferase | K02527 | 2.4.99.12, 2.4.99.13, | – | |

| Locus Tag | Gene | Gene Product | KO Number | EC Number | TC Number | HM resistance mechanism |
|---|---|---|---|---|---|---|
| | | | | 2.4.99.14, 2.4.99.15 | | |
| ABK249_03435 | *lptC* | lipopolysaccharide export system protein LptC | K11719 | – | 1.B.42.1 | |
| ABK249_03440 | *lptA* | lipopolysaccharide export system protein LptA | K09774 | – | 1.B.42.1 | |
| ABK249_03445 | *lptB* | lipopolysaccharide export system ATP-binding protein | K06861 | 7.5.2.5 | 3.A.1.152, 1.B.42.1 | |
| ABK249_05870 | *kdsD, kpsF* | arabinose-5-phosphate isomerase | K06041 | 5.3.1.13 | | |
| ABK249_10640 | *lptD* | LPS-assembly protein LptD | K04744 | – | 1.B.42.1 | |
| ABK249_10645 | *lptG* | lipopolysaccharide export system permease protein | K11720 | 7.5.2.5 | 3.A.1.152, 1.B.42.1 | |
| ABK249_10650 | *lptF* | lipopolysaccharide export system permease protein | K07091 | 7.5.2.5 | 3.A.1.152, 1.B.42.1 | |
| ABK249_10870, ABK249_12545 | *lpxL, htrB* | Kdo2-lipid IVA lauroyltransferase/acyltransferase | K02517 | 2.3.1.241, 2.3.1.- | – | |
| ABK249_16395 | *lpxC* | UDP-3-O-[3-hydroxymyristoyl] N-acetylglucosamine deacetylase | K02535 | 3.5.1.108 | – | |
| ABK249_20740 | *gmhA, lpcA* | D-sedoheptulose 7-phosphate isomerase | K03271 | 5.3.1.28 | – | |
| ABK249_23205 | *lpxD* | UDP-3-O-[3-hydroxymyristoyl] glucosamine N-acyltransferase | K02536 | 2.3.1.191 | – | |
| ABK249_23215 | *lpxA* | UDP-N-acetylglucosamine acyltransferase | K00677 | 2.3.1.129 | – | |
| ABK249_23220 | *lpxI* | UDP-2,3-diacylglucosamine hydrolase | K09949 | 3.6.1.54 | – | |
| ABK249_23225 | *lpxB* | lipid-A-disaccharide synthase | K00748 | 2.4.1.182 | – | |
| ABK249_23250 | *kdsA* | 2-dehydro-3-deoxyphosphooctonate aldolase (KDO 8-P synthase) | K01627 | 2.5.1.55 | – | |
| ABK249_24550 | *pagL* | lipid A 3-O-deacylase | K12976 | 3.1.1.- | – | |
| ABK249_27265 | *kdsB* | 3-deoxy-manno-octulosonate cytidylyltransferase (CMP-KDO synthetase) | K00979 | 2.7.7.38 | – | |
| ABK249_09240 | *fieF* | CDF family efflux transporter FieF | K13283* | – | 2.A.4.7.1 | Efflux outside the cell |
| ABK249_10420 | *atm1* | ATP-binding cassette, subfamily B, heavy metal transporter | K24821 | – | 3.A.1.210.3, 3.A.1.210.9, 3.A.1.210.11, 3.A.1.210.15 | |
| ABK249_12465, ABK249_21060 | *rcnB*-like | RcnB family regulator protein | K23243* | – | – | |
| ABK249_16755 | *rcnA* | Nickel/cobalt transporter (NicO) family protein | K08970 | – | 2.A.113 | |
| ABK249_22190 | *dmeF* | CDF family Co(II)/Ni(II) efflux transporter DmeF | – | – | 2.A.4.3.9 | |
| ABK249_22195 | *dmeR* | Ni(II)/Co(II)-sensing transcriptional repressor DmeR | – | – | – | |
| ABK249_01270, ABK249_09980, | *groES* | Chaperonin GroES | K04078 | – | – | Cytoplasmic sequestration |

| Locus Tag | Gene | Gene Product | KO Number | EC Number | TC Number | HM resistance mechanism |
|---|---|---|---|---|---|---|
| ABK249_14010, ABK249_29340, ABK249_29985 | | | | | | |
| ABK249_04850 | *ureE* | Urease accessory protein UreE | K03187 | – | – | |
| ABK249_06105 | *slyD* | FKBP-type peptidyl-prolyl *cis-trans* isomerase SlyD | K01802, K03775* | – | 5.2.1.8 | |
| ABK249_29950 | *hspA* | HSP20 family protein HspA | K13993 | – | – | |
| ABK249_13320 | *nikA*-like | Peptide/nickel transport system substrate-binding protein | K02035 | – | 3.A.1.5 | Periplasmic sequestration |

* KO identifiers assigned based on the results of a direct search in the KO database.
